# Breaking energy-power-stability trade-off in Li–S battery via HoMS

**DOI:** 10.1093/nsr/nwag382

**Published:** 2026-06-19

**Authors:** Yuanguo Wu, Xikun Zhang, Xingbao Zhu, Bao-Lian Su

**Affiliations:** Laboratory of Inorganic Materials Chemistry (CMI), University of Namur, Belgium; State Key Laboratory of Advanced Technology for Materials Synthesis and Processing, Wuhan University of Technology, China; School of Physics, Beijing Institute of Technology, China; Laboratory of Inorganic Materials Chemistry (CMI), University of Namur, Belgium; State Key Laboratory of Advanced Technology for Materials Synthesis and Processing, Wuhan University of Technology, China

Lithium–sulfur (Li–S) batteries are promising for next-generation sustainable energy storage, attributed to their ultrahigh theoretical energy density and the earth abundance and environmental benignity of sulfur. However, their cyclic stability and power density are severely compromised by the shuttle effect and the sluggish redox kinetics of lithium polysulfides (LiPSs) [1,2]. Different from lithium-ion batteries based on intercalation reactions, Li–S batteries based on conversion reactions typically suffer from an ‘impossible triangle’ among energy density, power density, and cycle life. Sluggish reaction kinetics leads to low power density, requiring catalysts to accelerate the redox reaction. However, catalysts provide no capacity and excessive catalysts lower the energy density, creating a trade-off between energy density and power density [3]. Meanwhile, long cycle life requires abundant adsorption sites to trap LiPSs and suppress the shuttle effect, but such sites rely heavily on excess host materials, which also reduces energy density, leading to another trade-off between cycle life and energy density. Current studies still lack effective solutions to this ‘impossible triangle’. Moreover, these problems are exponentially magnified when Li–S batteries shift from lab prototypes to scalable applications, directly hindering their practical implementation [4].

**Figure 1.**
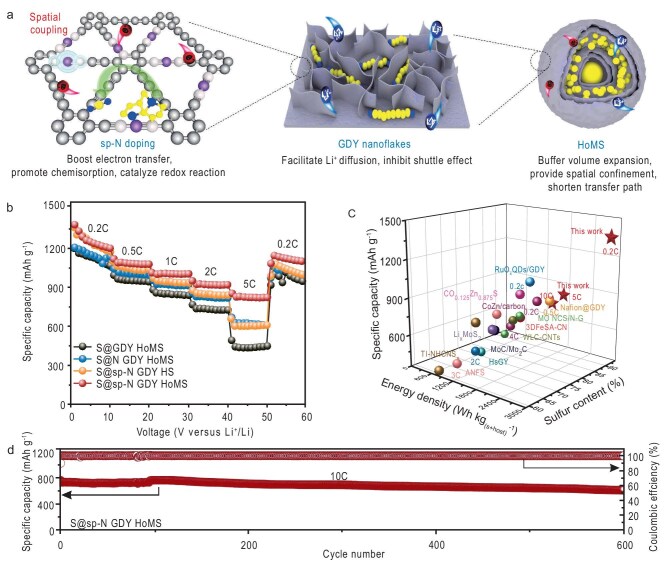
(a) Scheme showing the advantages of S@sp-N GDY HoMS cathode. (b) Rate performance of Li–S batteries based on different sulfur cathodes. (c) Comparison of specific capacities at different current densities with reported Li–S batteries. (d) Long cycling performance of S@sp-N GDY HoMS cathodes at 10C. Adapted with permission from Ref. [5].

To resolve this long-standing dilemma, recently, Wang and co-workers proposed a groundbreaking design paradigm: spatial coupling of adsorption and catalytic sites within a *sp*-nitrogen-doped graphdiyne (*sp*-N GDY) hollow multishelled structure (HoMS), thereby integrating the functionalities of host and catalyst into a single material to minimize inactive mass [5]. GDY, which was first reported by Li and co-workers [6], possesses a unique *sp^2^*–*sp* hybridized carbon skeleton and offers inherent doping versatility. Wang and co-workers previously reported a new *sp*-N doping into GDY flakes for the first time through a pericyclic reaction [7] and pioneered the construction of *sp*-N GDY HoMS in this recent work (Fig. 1a) [5]. The favorable orbital overlap between *sp*-N atoms and adjacent carbon atoms facilitates electron transfer and optimized charge redistribution. Consequently, the negatively charged *sp*-N atoms and positively charged neighboring carbon atoms act as spatially coupled adsorption and catalytic sites, realizing a closed loop of ‘LiPSs spatial confinement—*in situ* catalytic conversion’, synergistically promoting both LiPSs adsorption and redox conversion. Additionally, HoMS constructed from GDY nanoflakes enhances lithium ion diffusion and provides multiple spatial confinement effects to suppress LiPSs shuttling.

Notably, with an ultrahigh sulfur loading of 93.9%, the *sp*-N GDY HoMS-supported sulfur (S@sp-N GDY HoMS) cathode delivers a near-theoretical capacity of 1462 mAh g_(S+host)_⁻¹ at 0.1C (Fig. 1b). More impressively, it achieves a record-high energy density of 1384.5 Wh kg_(S+host)_⁻¹ and power density of 29 457.4 W kg_(S+host)_⁻¹ at 10C, while sustaining stable cycling for over 600 cycles (Fig. 1d). The practical viability of this design is further validated by pouch cells, which exhibit an energy density of ∼457 Wh kg⁻¹, far surpassing commercial lithium-ion batteries (Fig. 1c). A comprehensive suite of in situ characterizations directly confirms the efficient adsorption and rapid conversion of LiPSs by the *sp*-N GDY HoMS. Density functional theory calculations further validate the synergistic adsorption-catalysis effect.

The findings reported by Wang and co-workers make seminal contributions to the Li–S battery field by breaking the decades-long ‘impossible triangle’ bottleneck that plagued energy density, power density, and cyclic stability simultaneously. This work effectively eliminates the core technical obstacle for Li–S batteries transitioning from laboratory prototypes to industrial deployment, thereby strongly advancing the practical application of high-performance Li–S batteries. Furthermore, this design principle of spatially coupled dual active sites provides a universal design paradigm for advanced battery systems based on conversion reactions (e.g. Na–S batteries, metal–air batteries, etc.), which universally suffer from the same ‘impossible triangle’ dilemma. Moreover, this China-originated *sp*-N GDY HoMS with spatially coupled dual active sites not only greatly reduces the technical threshold and cost but also holds great promise for broader applications in energy conversion and storage, further spurring technological innovation toward global carbon neutrality.
